# An Auditory Neural Correlate Suggests a Mechanism Underlying Holistic Pitch Perception

**DOI:** 10.1371/journal.pone.0000369

**Published:** 2007-04-11

**Authors:** Daryl Wile, Evan Balaban

**Affiliations:** 1 Behavioral Neurosciences Program, McGill University, Montreal, Canada; 2 Cognitive Neuroscience Sector, Scuola Internazionale Superiore di Studi Avanzati (SISSA), Trieste, Italy; Indiana University, United States of America

## Abstract

Current theories of auditory pitch perception propose that cochlear place (spectral) and activity timing pattern (temporal) information are somehow combined within the brain to produce holistic pitch percepts, yet the neural mechanisms for integrating these two kinds of information remain obscure. To examine this process in more detail, stimuli made up of three pure tones whose components are individually resolved by the peripheral auditory system, but that nonetheless elicit a holistic, “missing fundamental” pitch percept, were played to human listeners. A technique was used to separate neural timing activity related to individual components of the tone complexes from timing activity related to an emergent feature of the complex (the envelope), and the region of the tonotopic map where information could originate from was simultaneously restricted by masking noise. Pitch percepts were mirrored to a very high degree by a simple combination of component-related and envelope-related neural responses with similar timing that originate within higher-frequency regions of the tonotopic map where stimulus components interact. These results suggest a coding scheme for holistic pitches whereby limited regions of the tonotopic map (spectral places) carrying envelope- and component-related activity with similar timing patterns selectively provide a key source of neural pitch information. A similar mechanism of integration between local and emergent object properties may contribute to holistic percepts in a variety of sensory systems.

## Introduction

Pitch, a subjective attribute of auditory stimuli which allows sounds to be arranged on a scale from low to high, is a salient component of most natural and artificial sounds, including speech and music [1]. Its derivation by brains has sparked controversy ever since the 1840s when Georg Ohm first proposed that the ear works as a Fourier analyzer, and August Seebeck presented a discrepant phenomenon: a stimulus with a severely attenuated lowest component is subjectively assigned the same pitch as one with the lowest component at full strength (the “missing fundamental” pitch percept) [2]. Many models have since been proposed to account for the holistic nature of pitch percepts [3], focusing either on neural timing patterns or spatial patterns of neural activation extracted over the whole array of auditory frequencies. However, neither type of theory has proven able to explain all pitch perceptual phenomena, leading to a search for mechanisms combining these two kinds of information. The present work examines whether brain pitch calculations may entail a combination of two different types of neurally-coded timing information originating at common places within the brain's tonotopic map.

**Figure 1:**
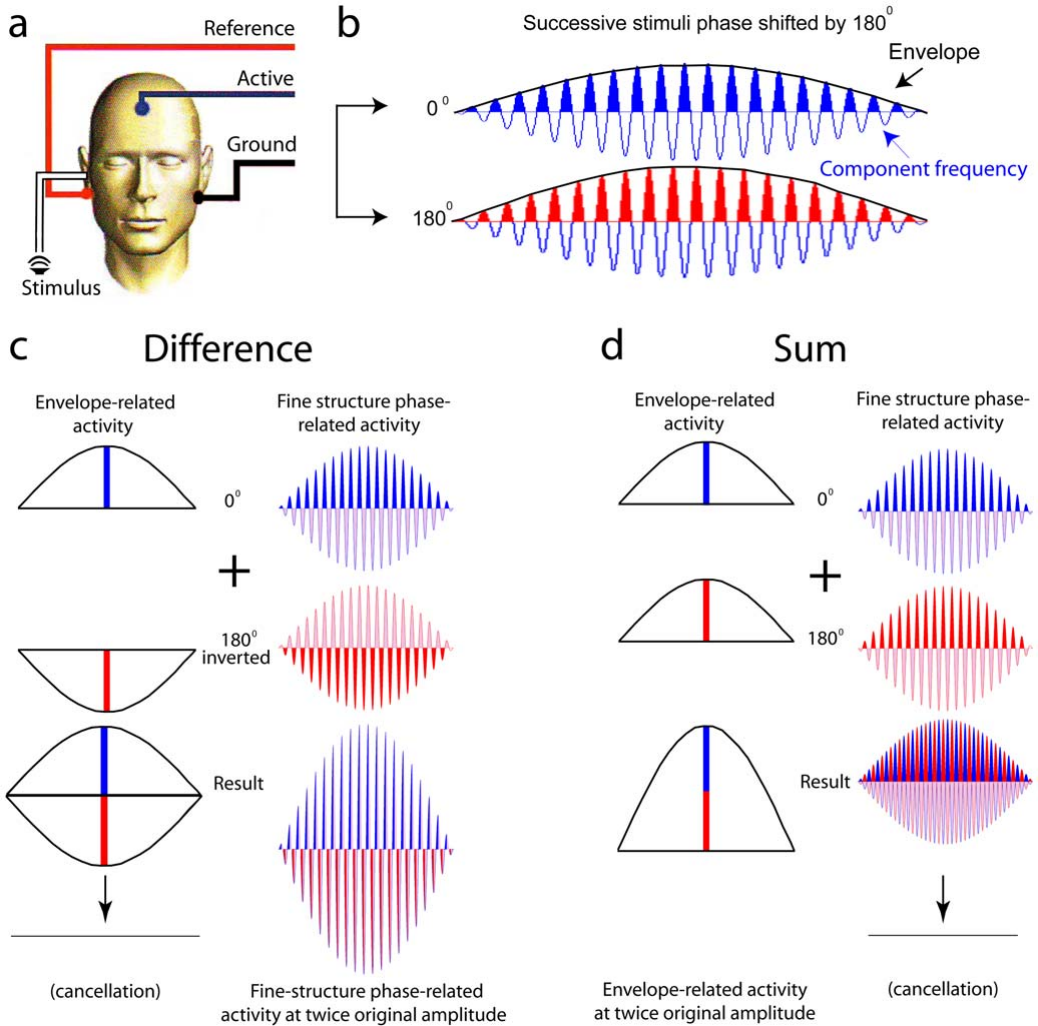
Experimental design. (a) Schematic location of stimulus presentation and recording sites on the scalp. (b) Successive stimuli were phase shifted by 180°. This moves the locations of maxima and minima by ½ cycle relative to their unshifted values, leaving the envelope (black) unchanged but changing the relative location of maxima and minima in the component frequencies (the fine-structure, blue and red). (c) Difference waveform generated by multiplying the 180° response waveform by −1 and adding it to the 0° response waveform. Since the envelope-related activity near the maxima of the 180° waveform (red) now has the opposite sign from that of the 0° waveform (blue), these two cancel out; fine-structure-related activity now has the same sign for the 180° and 0° waveforms, yielding double the original signal strength. (d) Sum waveform generated by adding the 0° response waveform to the 180° response waveform. Envelope-related activity near the maxima now have the same sign (blue and red), yielding double the original signal strength; fine-structure-related activity now has opposite signs for the 0° and 180° response waveforms, canceling each other out.

Humans and other mammals display highly similar holistic pitch percepts, assigning a low-frequency pitch to sounds that have spaced, tonal higher-frequency components, even when low-frequency components are absent (the missing fundamental) [4], [5]. Despite the recent discovery of primate auditory cortical neurons that appear to be selective for holistic pitch [6], there is no clear picture of how information converges within the auditory pathways that contribute to the response characteristics of these neurons. Precise subjective reports of holistic pitch percepts are laborious to implement in animal subjects, but can be easily measured in humans by adjusting the frequency of a pure tone until it matches the apparent pitch of a stimulus sound. The present work examines such perceptual data collected from 22 normally-hearing human participants in relation to an auditory-evoked electrical potential with a broad topographic distribution on the scalp called the frequency-following response (FFR) [7]–[9], thought to originate in the auditory brainstem and midbrain [10]–[12]. This signal was not chosen to elucidate the role these brain regions play in pitch perception, but rather because information relevant to pitch still exists in a temporal code at the FFR stage. The relative strengths of different temporal subcomponents can be extracted and directly compared, a process that would be difficult once this information is converted to a rate-place code at subsequent stages of brain processing. The present study focuses on this ability to measure the relative strengths of different components of the FFR rather than on its site(s) of origin (Figure 1, Figure S1).

**Figure 2:**
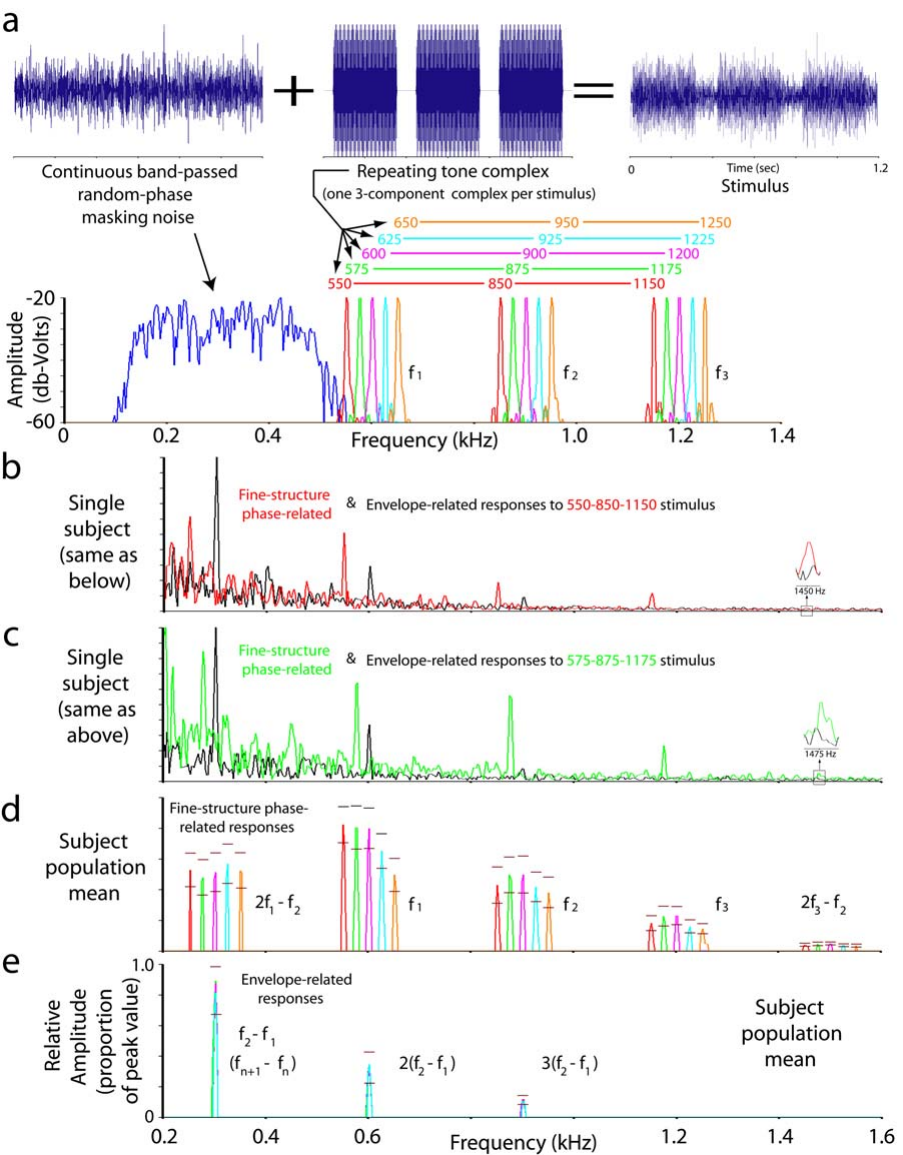
Stimulus design and evoked potential responses. (a) Amplitude-time waveforms showing stimulus construction by adding band-passed, random phase noise (top, left) to repeating 3-note tone complexes (top, center) to produce the stimuli used in perceptual testing and for evoked-potential recording. There were five different tone complexes used; each is shown color-coded above its constituent components on an amplitude–frequency plot (bottom), where the components of each tone are overlaid on a common set of axes. (b,c) Amplitude spectra of the evoked potential responses from a single subject for two of the stimulus tones. The sum of the 0° and 180° responses is shown in black (envelope-related), and the difference of the 0° and 180° responses is shown in red or green (fine-structure phase-related). The y-axis is in units of relative amplitude; the corresponding measured values for the highest y-axis marker are 0.38 µV (black) and 0.35 µV (red) for Figure 2b, and 0.26 µV (black) and 0.23 µV (green) for Figure 2c. Colors refer to Figure 2a. (d,e) Mean relative amplitude spectra (n = 22 subjects) of peaks in the fine-structure-related (difference, Figure 2d) and envelope-related (sum, Figure 2e) evoked potential responses, all of which passed criteria for robustness (see Methods). Responses to each stimulus tone have been overlaid on the same set of axes. Colors refer to Figure 2a. Error bars represent 95% confidence intervals around the mean value.

Like most natural sounds, the stimulus tone complexes used here are characterized by rapidly-varying amplitude oscillations of each individual frequency component (the component-specific “fine structure”) together with a more slowly-varying amplitude oscillation of the envelope of the composite waveform (an emergent feature of the tone complex). Auditory nerve fibers exhibit activity entrainment to both of these classes of timing intervals, concentrating their activity around the maximum of amplitude that occurs with each respective cycle (called phase-locking) [5]. By recording responses to the same stimulus successively phase-shifted by 180° [13], [14], Figure 1 illustrates how component-specific, fine-structure-related and holistic, envelope-related components could be separately obtained from the FFR. This technique was used to examine the relationship between temporal activity patterns of component-related and envelope-related neural activity during holistic pitch perception. By simultaneously presenting low-frequency masking noise (Figure 2a,b), it was possible to limit the regions of the tonotopic map that conveyed this information centrally.

## Results

The experiments used 5 different stimulus tone complexes (one with harmonically-related components that are integer multiples of each other, and 4 frequency-shifted, inharmonic versions of this same complex). Because frequency-shifting the tone complexes leaves the distance between tones unaltered but changes their relative position on the tonotopic map, all of these stimuli have identical envelopes, but differ in the frequencies of their tonal components and in their pitch percepts [1]. Brain activity that is phase-locked to the individual components should shift in frequency according to the frequency shift of the components, while activity that is phase-locked to the envelope should remain at the same frequency for all stimuli. Using the method illustrated in Figure 1, we found a set of well-separated frequency peaks in Fourier spectra of the fine-structure-related and envelope-related brain response waveforms whose presence and magnitude were robust among subjects (Materials and Methods, Figure 2, Text S1, Figure S1). These peaks represent temporal rates of neural activity with local energy maxima phase-locked to the fine structure of the tonal components, and to the envelope of the stimulus. No robust, consistent peaks that shifted frequency in parallel with tone complex shifts were observed in the vicinity of the missing fundamental pitch percepts of the individual participants, a possibility suggested by preliminary data on a single subject in a previous study [8].

There were two different kinds of fine-structure-related frequency peaks (Figure 2d): those at each of the 3 “primary” tone frequencies (f_1_, f_2_ and f_3_), and so-called “distortion products”, produced at a spacing of 300 Hz below f_1_ (2f_1_-f_2_ or 2f_n_ - f_n+1_) [15]–[19] and 300 Hz above f_3_ (2f_3_ – f_2_). The fact that these peaks were all frequency-shifted along with the stimuli demonstrates that they all resulted from activity that was phase-locked to the fine-structure [14], [20]. The distortion products are thought to result from processes responsible for cochlear amplification control [17], manifested in two separable ways: (i) a “generative” component produced by interactions of the stimulus frequencies within spatially-limited regions of the cochlea at frequencies above f_1_, and incorporated into nerve firing patterns within those regions, and (ii) a “propagated” component that travels along the basilar membrane as a result of the energy produced by the generative component, stimulating hair cells at the appropriate frequencies 300 Hz below f_1_, and 300 Hz above f_3_. Perception of individual distortion products is blocked by masking noise centered at their apparent frequency [21], but unitary missing fundamental pitch percepts persist in the presence of such noise [22]. Because all of the data presented here were collected in the presence of masking noise sufficient to block activity related to low-frequency propagated components (Figure 2a,b), these cannot play a role in the present results. Envelope-related activity occurred with the same pattern for all 5 stimuli, with peaks at the envelope and its first two harmonic frequencies (Figure 2e).

**Figure 3:**
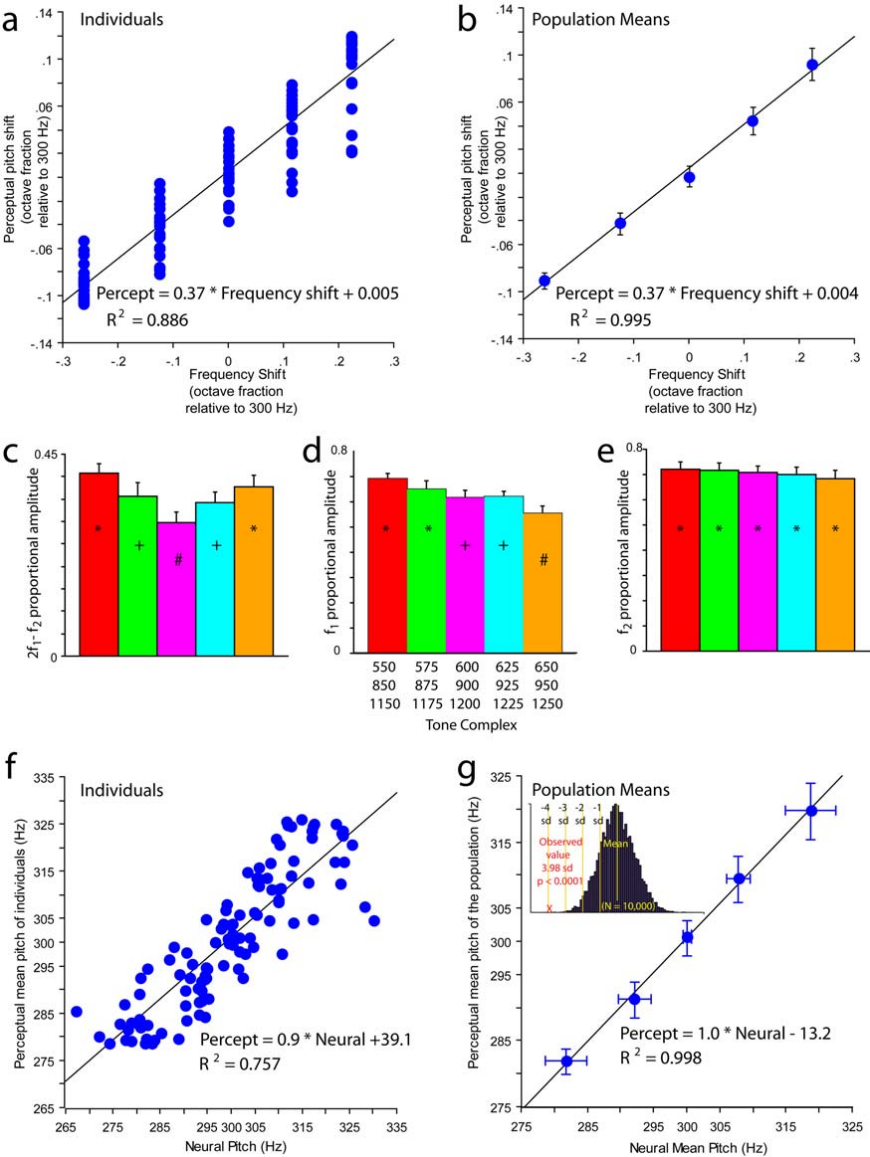
Pitch percepts and their relationship to evoked potential responses. (a) Mean pitch percept (10 repetitions) of each stimulus for each of 22 subjects plotted against the frequency shift of each stimulus; units are octave fractions relative to 300 Hz. The equation of the best-fitting regression line and the r^2^ are also shown. (b) Population mean pitch percepts (22 subjects) plotted against the frequency shift of each stimulus, units as in (a). The error bars show 95% confidence intervals around the mean values. (c,d,e) The mean values (22 subjects) for the proportional amplitude of the difference waveform (fine-structure-phase-related) spectral peaks for the 5 stimuli. The error bars show 1 standard error around the mean values. Bars containing the same symbol are not significantly different from each other; bars containing different symbols are significantly different from each other (see Methods). Colors refer to Figure 2a. (f) Mean pitch percept of each stimulus for each of 22 subjects plotted against their own neural pitch estimates. The equation of the best-fitting regression line and the r^2^ are also shown. (g) Population mean pitch percepts (22 subjects) plotted against the population mean neural pitch estimate. The error bars show 95% confidence intervals around the mean values for each axis. (Inset) The frequency distribution of distances of population neural pitch estimates from the population pitch percept according to a random model (black) compared with the distance obtained in this study (red cross), showing that the present results are highly unlikely to be obtained by chance (see Methods).

Perceptually, the 22 participants performed similarly to those described in previous experiments, with their pitch percepts conforming to ‘de Boer's Rule’ [23], [24], which predicts a slope for the regression of percept on frequency shift slightly greater than 1/3 for these stimuli (Figure 3a,b). This regression accounted for 88.6% of the variation in pitch percepts of individual participants (df = 1,108; F = 840.6, p<0.0001, Figure 3a), and 99.5% of the variation in the mean performance averaged across all participants (df = 1,3; F = 1596.1, p<0.0001, Figure 3b).

The pattern of variation seen in Figures 2d and 2e, with envelope- and fine-structure-related peaks occurring close to each other in each of 3 separated bands of frequencies (∼300 Hz, ∼600 Hz, ∼900 Hz), suggested the potential for information from envelope and fine-structural components to interact centrally within the brain. A simple way for this to happen would be for neural circuitry to derive a weighted average of the frequencies at envelope and fine-structure peaks that are within the same half-octave, according to the relative amounts of activity (reflected by the height of the peaks) at each of their respective frequencies. For example, if the proportion of the joint responses represented by envelope activity at 300 Hz and fine-structure activity at 250 Hz given in response to a 550–850–1150 Hz tone complex were 0.58 and 0.42, respectively, this operation would compute the sum (0.58*300 Hz)+(0.42*250 Hz) = 279 Hz. Since the 300 and 250 Hz activity peaks are produced by populations of neurons getting information from the same region(s) of the basilar membrane, this would represent a joint coding scheme whereby the places of joint activation determine which timing-related signals get combined together. Such a weighting system could quantitatively account for the roughly symmetrical size of the changes in observed pitch percepts with positive and negative linear frequency shifts (Figure 3a,b) only if the proportion of activity due to the part-related, fine-structural component increased quasi-symmetrically as the value of the frequency shift moved away from zero, producing a roughly U or V-shaped curve. This would cause the weighted average to increasingly diverge from the midpoint between the two frequencies as the shift got larger.

Figures 3c,d,e show the variation of the relative proportion of response amplitude accounted for by fine-structure components. The 2f_1_–f_2_ fine-structure component (near the 300 Hz envelope component) has a U or V-shaped pattern of proportional amplitude variation (Kruskal-Wallis One-Way ANOVA, H = 11.55, df = 4, p = 0.02, Figure 3c); successive frequency-shifted tones have proportional amplitude values that are significantly different from one another. This results from significant changes in the peak amplitudes of the 2f_1_–f_2_ component with frequency shift (Kruskal-Wallis One-Way ANOVA, H = 10.12, df = 4, p = 0.038) superimposed on non-significant changes in the peak amplitudes of the 300 Hz envelope component with frequency shift (Kruskal-Wallis One-Way ANOVA, H = 1.05, df = 4, p = 0.90). In contrast, the f_1_ component (near the 600 Hz first harmonic of the envelope) also changes with frequency shifts (Figure 3d, Kruskal-Wallis One-Way ANOVA, H = 13.25, df = 4, p = 0.01), but successive frequency shifted values are not significantly different from each other, and the pattern is anti-symmetrical rather than symmetrical about zero. The f_2_ component (near the 900 Hz second harmonic of the envelope) exhibits no such significant changes (Figure 3e, Kruskal-Wallis One-Way ANOVA, H = 1.00, df = 4, p = 0.91).

Figure 3f and g show the neural pitch predictions produced by the combination of the 2f_1_–f_2_ fine-structural component and the 300 Hz envelope component, plotted against the perceptual data from the same subjects. The neural predictions explain about 76% of the variation in individual pitch percepts (Figure 3f, df = 1,108; F = 336.3, p<0.0001); for population data, the neural pitch predictions explain about 99.8% of the variation in subjective percepts with a regression line slope of 1.0 (Figure 3g, df = 1,3; F = 1614.0, p<0.0001). Such a close correspondence between neural pitch predictions and subjective pitch percepts cannot be accounted for by chance (inset to Figure 3g).

## Discussion

This study found that holistic pitch perception is very strongly mirrored by a simple activity-weighted combination of fine-structure-related and envelope-related brain activity recorded at the level of the brainstem and midbrain, resulting from the activation of spectrally-limited higher-frequency regions of the cochlea. We propose that holistic pitch perception of tonal sounds involves a place-gated combination of neural timing information from the envelope and fine-structure of a sound relayed via the midbrain and brainstem; the lowest region of the tonotopic map in which envelope and fine-structural components have similar timing patterns dynamically assumes a dominant role in pitch processing. An operation analogous to a weighted average of these timing patterns is performed, based on the relative amounts of activity in the fine-structural and envelope components. Since temporal information at the missing fundamental pitch is absent from the FFR, this operation would most plausibly be calculated after the conversion of auditory-related activity from a temporal to a rate-place representation, as a spatial average of tonotopic rate-place representations of fine-structure and envelope information.

The present work has two notable features. It is, to our knowledge, the first demonstration linking subjective pitch percepts to a quantitative combination of envelope- and fine-structure-related neural activity, although earlier perceptual work indirectly indicated some kind of relationship between envelope- and fine-structural features in pitch perception [25]. Second, the fine-structural neural activity it implicates in pitch perception stems from distortion products that are the result of non-linear dynamics during the sensory transduction process in the inner ear [26], [27], [28]. In regions of the basilar membrane that are sensitive to more than one frequency component present in the stimulus, the cochlear amplification process results in neural activity at distortion product frequencies [28]. We predict that interfering with 2f_1_–f_2_ neural activity at the site of its generation should disrupt missing fundamental pitch percepts, and that the frequency ‘region of existence’ over which missing fundamental pitch percepts are generated may be explained by the size of the region in which phase-locked 2f_1_–f_2_ distortion products are generated, and the strength with which they are generated. We hope to test these predictions in future work.

It has recently been proposed that single neurons in the lower auditory central nervous system generate spike trains at missing fundamental frequencies via a nonlinear “ghost” stochastic resonance mechanism [29], [30]. Our failure to find FFR activity at missing fundamental frequencies does not necessarily contradict this hypothesis (the signal sources may not be large enough to be detected, they may be spatially organized in such a way that they are not well reflected in EEG signals, and such neurons may be outside of the networks that contribute to FFR generation). The stochastic resonance mechanism, while physiologically plausible, has not yet received empirical verification. The model proposed here explains similar phenomena with reference to empirically- known properties of auditory neurons.

What mechanisms are responsible for the observed shifts in the proportional amplitude of 2f_1_ –f_2_ component-related activity with frequency shifts in the tone complexes? Single auditory nerve fibers show a decreasing 2f_1_ –f_2_ response as the frequencies of two stimulating tones get further apart [15], [16], [28]. This phenomenon could explain the response to upward tone-complex shifts observed here, since the spacing of linearly-shifted components decreases relative to the logarithmic scale of the tonotopic frequency map. However, this would not account for increases in response strength observed with downward linear shifts. A possible explanation is based on the sharp slope of high-frequency cutoffs and shallow slope of the low-frequency tails of auditory fiber frequency responses. As a tone complex with more than two components is linearly shifted downward by a small amount, it may gain more response strength in terms of fibers responding to multiple tone components than it loses, even though the relative spacing between tones increases. However, once the distance between tone components becomes sufficiently large, the number of fibers that respond to multiple components would start to decrease, leading to a decline in the activity contributing to holistic pitch percepts. Further research is required to more fully evaluate these ideas.

This work may also have more general implications for processing object attributes in the auditory, visual and somatosensory systems, where many brain regions maintain the topographic organization of information arriving from the sensory periphery. The broader topographic spatial distribution of whole-object-related patterns of neural activity (like the envelope studied here) may set up different kinds of interactions with object-part-related information in different regions of a topographic map, and, singly or in combination, these could contribute to different aspects of perception. For example, timbre, another holistic attribute of sounds, may be derived in part from integrative comparisons between envelope and fine-structural information over a larger region of the tonotopic map than is used for holistic pitch. It may prove fruitful to re-examine basic patterns of part-related and whole-object-related activity encoded by nervous systems that the brain may recombine in different ways to produce subjectively-independent holistic attributes of our experiences.

## Materials and Methods

### Participants

Twenty-eight participants were recruited from the McGill University community (18 females), with no history of central or peripheral auditory damage. Testing of each participant was completed consecutively on one day, and all participants either received extra credit points for an undergraduate psychology course or were unpaid volunteers. All experimental protocols were reviewed and approved by the McGill Research Ethics Board (REB-II). Before examining any evoked potential data, linear regressions were calculated for each participant between the mean pitch percept (over ten repetitions) for each stimulus and the frequency shift of the stimuli (as in Figure 3a,b), in order to retain only participants with robust and consistent pitch percepts in subsequent analyses. We adopted the criterion of a linear regression with an r^2^ value of ≥0.95 in order for a participant to be included; 22 (14 females) of the 28 participants reached this criterion and were used for all analyses presented in this report. The excluded participants had a mean r^2^ value of 0.76 and non-linear plots unlike the linear plots found in previous studies (and for the included participants here). We believe the excluded participants either did not understand the task correctly and/or did not give themselves enough practice with the training stimuli. On subsequent examination, the neural data of the excluded participants exhibited the same general features shown by the included ones.

### Stimulus generation and delivery

Complex tone and noise stimuli were generated using SIGNAL digital signal analysis language (Engineering Design, Berkeley CA, USA). All tone complexes contained three components in 0° (sine) phase that were equal in level. Stimuli in both the psychophysical and evoked potential experiments were presented through the right ear using an E·A·RTone 3A insert tube earphone (E·A·R Auditory Systems, Indianapolis IN, USA) coupled to a foam ear insert.

Band-passed masking noise was also generated for playback through the insert earphone at a level 10 dB below the primaries to mask propagated basilar-membrane distortion products within the region of the missing fundamental for the stimuli; this was produced by filtering white noise between 150–450 Hz with a 100 dB/octave attenuation rate at the shoulders. In the psychophysical experiment, random-phase noise was generated together with each stimulus playback and was of the same duration as the tone complex stimulus (1 s, including 5 ms cosine^2^ rise/fall times). In the evoked potential experiments, tone complex stimuli were played for a duration of 310 ms (including 5 ms cosine^2^ rise/fall times) with 45 ms of silence before and after the stimulus (an overall window duration of 400 ms, resulting in a 90 ms silent gap between successive repeats of the tone complexes). A second channel feeding into the single E·A·RTone 3A insert earphone played the cycling band-passed masking noise window of duration 300.25 ms with no silent gap, such that the tone complex and the band-passed masking noise were repeating at different non-integer time intervals, allowing the noise to have a randomized phase with respect to the tones. In this way, phase-coherent responses to the tone could be averaged without averaging phase-coherent responses to the noise.

All stimuli in both the psychophysical and evoked potential experiments were presented at an overall level of 74.5 dB(A) as measured through the insert earphone coupled to a sound level meter (Bruel and Kjaer 2209, Naerum, DK) via the 2 cm^3^ coupler of an artificial ear (Bruel & Kjaer 4152). The levels of distortion products generated within the stimulus delivery system were measured empirically (2f_1_-f_2_: 35–37 dB below the level of f_1_; f_2_-f_1_: 45–48 dB below the level of f_1_); these low-frequency distortion products were completely masked by the band-passed noise in the region of 200–400 Hz (10 dB below the level of f_1_).

### Data collection procedures

All experiments took place in an electrically isolated, double-walled sound-attenuating chamber (Industrial Acoustics Model 1202, New York NY, USA). Psychophysical pitch-matching data were obtained with a laptop computer running a computer program written in the SIGNAL language. Psychophysical test stimuli were played back (40 kHz sampling rate) using a digital-to-analog converter (National Instruments DAQCard 6062-E, Austin TX, USA). Evoked potential recordings used the same stimuli played back through the SmartEP Evoked Potential System (Intelligent Hearing Systems, Miami FL, USA).

To ascertain the perceived pitch of all complex tone stimuli, participants adjusted the frequency of a pure tone to match the test complexes. The participant-adjusted pure tone and the test complex were embedded in sequences of four tones which participants could repeatedly listen to while adjusting the frequency of the pure tone (or leaving it the same if unsure whether the tones were correctly matched) between presentations. The sequence also contained a reference harmonic tone complex composed of 440, 660 and 880 Hz components equal in level and duration to those of the test complex, and a reference pure tone of frequency 220 Hz which was therefore matched in pitch to the reference tone complex. The order of tones in the stimulus sequence was: reference tone complex, test complex, reference pure tone, participant-adjusted pure tone. The frequencies of the reference tones remained constant throughout the experiment, and both the test complex and the reference tone complex contained masking noise as described above. This design provided extra cues in the form of matching the pitch interval information between the first and second (tone complex) and third and fourth (pure) tones. Participants were instructed to match the pitch of the second pure tone to the “overall” or synthetic pitch of the second tone complex.

Prior to commencing the psychophysical experiment, the pitch-matching paradigm was explained and participants completed a training session. In this training session, participants made pitch matches to five random *f*_0_ harmonic tone complexes (250 Hz<*f*_0_<350 Hz). Participants could freely adjust the value of the pure tone and listen to the result as many times as they liked. When satisfied that the adjusted pure tone was matched to the tone complex, participants submitted their final estimate and received as feedback the true fundamental frequency of the test complex. Participants were able to repeat the training session if they wanted additional practice. The frequency of the participant-adjusted pure tone was initially set to a random value between 235 and 245 Hz, after which it retained its previous values repeatedly reset by the participant over the course of one trial.

After the training session was complete, participants completed the psychophysical experiment, which included 10 presentations of each stimulus, as well as 10 random *f*_0_ harmonic tone complexes (250 Hz<*f*_0_<350 Hz), in random order. As in the training session, participants could freely adjust the value of the pure tone and listen to the result as many times as they liked before submitting a final estimate. Breaks were available to participants during the experiment if necessary. No feedback was given to participants about their pitch matches during the experiment. The frequencies of the reference tones and the initial randomization of the participant-adjusted pure tone at the beginning of each trial were the same as in the training session.

Following the psychophysical experiment, all computing equipment was removed from the sound-attenuating chamber, participants were seated in a reclining chair, and prepared for recording of evoked potentials. Preparation involved light abrasion of the skin on the left and right mastoids and the forehead (at FPz). Conductive adhesive Ag/AgCl electrodes (Kendall Meditrace 133, Chicopee MA, USA) were attached according to the 10–20 international electrode placement system, with the active electrode on the forehead (FPz), the reference electrode on the right mastoid (M2) and the ground electrode on the left mastoid (M1). Electrode impedances were less than 3 kΩ for all recordings. Electrophysiological data were acquired using the SmartEP Evoked Potential system. Signals were sampled at 10 kHz and amplified 100,000 times using an optically-coupled amplifier (Intelligent Hearing Systems). With these settings, empirical measurements of multicomponent electrical signals with similar frequencies to the stimuli and in the same voltage range as the recorded brain potentials yielded no evidence of distortion products, indicating that any intermodulation distortion produced by the amplifier is below the level of the noise floor of the recordings. Responses were band-pass filtered from 100 to 1500 Hz, and samples containing voltages exceeding 31 µV were rejected by an automatic artifact rejection utility. Stimulus polarity alternated on each sweep, and alternate-polarity sweeps were recorded and averaged in separate onboard data buffers by the SmartEP system. During stimulus presentation, participants were asked to lie in a restful state and told to refrain from moving as much as possible; 2048 non-rejected sweeps (1024 for each polarity) were recorded for each stimulus. Control recordings in which all of the same procedures were followed with the tubes of the insert earphones blocked resulted in no signal energy above the noise floor at stimulus component, envelope or distortion product frequencies.

### Data analysis

Perceptual data were analyzed by taking the mean of the 10 pitch judgments made in response to each stimulus for each participant, and using these as the primary data characterizing pitch percepts, resulting in 110 total percepts (22 participants, 5 stimulus tones). Means across the 22 participants for each stimulus were also calculated to characterize the behavior of the participant population. Linear regression and Kruskal-Wallis One-Way ANOVA statistics were calculated in Statview (SAS Institute, Cary NC); post-hoc significance tests for the Kruskal-Wallis One-Way ANOVA were carried out by the procedure described [31] with a p<0.05 level of significance.

The 0°and 180° response waveforms from the SmartEP system were transferred to SIGNAL and MATLAB (The Mathworks, Natick MA) for further analysis. Sum and difference waveforms were generated for each participant and stimulus as described in Figure 1; amplitude spectra for these sum and difference waveforms were generated from the waveform segment beginning 50 msec after the onset of the stimulus (to allow for the stabilization of brain responses) to the end of the stimulus window (4096-point zero-padded Discrete Fourier Transform, Hanning window). To set a criterion for the robustness of peaks for acceptance in subsequent analyses, the values in each individual amplitude spectrum were expressed as a proportion of the maximum value between 0–4000 Hz in that amplitude spectrum, and spectra were rms-averaged across all 22 participants for each stimulus type. This average was used to calculate a standard deviation spectrum for each stimulus type. The standard deviation spectrum was smoothed with a 200-Hz window to produce an estimate of the standard deviation of the noise floor of the spectrum. This value was then turned into an upper 99% confidence limit for the noise floor according to a two-tailed t-distribution (df = 21). Peaks exceeding this criterion were considered to be robust among participants. For all five stimulus tones, all peaks shown in the lowest two panels of Figure 2 met this criterion.

Neural pitch estimates were generated by extracting the frequency and amplitude values of the components shown in the lowest two panels of Figure 2, using a search window 20 Hz wide centered on each component of interest. The local maximum within this window with the highest value was selected, and its frequency and amplitude were recorded (results were no different for search windows between 10 Hz and 30 Hz wide). This yielded one set of frequency and amplitude values for the sum and difference spectral components for each stimulus for each subject. Neural pitch predictions for each subject were calculated by summing the amplitude values of the lowest sum-waveform and difference-waveform peak above [f_1_-330] Hz for each stimulus, and dividing each peak value by this sum to obtain a proportion of the response amplitude due to the 2f_1_-f_2_ (lowest difference-waveform) spectral component and the envelope (lowest sum-waveform) spectral component. Predicted pitches were the average of the two frequencies weighted by the relative strength of their responses. To calculate the probability of obtaining the observed result by chance, this analysis was repeated using amplitudes picked at random (uniform random distribution) from between the highest and lowest values obtained in the data set for each participant, and frequencies picked at random (uniform random distribution) within each of the 20-Hz wide windows used for the difference and sum spectra in the data analysis. Each iteration of the probability calculation generated these numbers for 22 fictive participants for each of the five stimuli, used these to generate “neural pitches” as above, took the mean of these over the 22 fictive participants for each stimulus tone, and calculated the distance of this mean neural pitch estimate (absolute value of [neural pitch–subjective pitch], summed over all five stimuli). Ten thousand iterations were performed. The mean distance of the random expectation was 19.97 Hz, the minimum distance was 7.14 Hz and the maximum distance was 35.44 Hz, compared with the observed value of 4.22 Hz, resulting in a probability of<0.0001 of obtaining the observed results by chance (Figure 3).

## Supporting Information

Text S1 (0.04 MB DOC)

Figure S1 Mean evoked potential responses to stimuli. (a) The five different tone complexes used are shown color-coded above their constituent components on an amplitude - frequency plot (bottom), where the components of each tone are overlaid on a common set of axes. (b,c) Mean relative amplitude spectra (22 participants) for the 0° (in b) and 180° (in c) responses, showing the similarity in the response patterns evoked by these stimulus classes. Responses to each stimulus tone have been overlaid on the same set of axes. Colors refer to part (a) of the figure. (d,e) Mean relative amplitude spectra (n = 22 participants) of peaks in the fine-structure-related (difference waveform, d) and envelope-related (sum waveform, e) evoked potential responses. Responses to each stimulus tone have been overlaid on the same set of axes. Colors refer to part (a) of the figure.
(2.27 MB TIF)
