# Supplementary material for: An Auditory Neural Correlate Suggests a Mechanism Underlying Holistic Pitch Perception
Source: PLoS One. 2007 Apr 11;2(4):e369. doi: 10.1371/journal.pone.0000369 (PMC1838520; doi:10.1371/journal.pone.0000369)
Supplement: Text S1 — (0.04 MB DOC) [file pone.0000369.s001.doc]

**Supporting Information**

**The role of cochlear microphonics**

One potential complication for the present experiments is the extent to which response recordings were contaminated by cochlear microphonic potentials, which may be present in the envelope-related brain activity waveform derived from the sum of the 0° and 180° evoked potential waveforms [S1, S2]. We did not see peaks at 300, 600 or 900 Hz in the difference waveforms from stimuli with nonzero frequency shifts, suggesting that such potentials, if present, were effectively cancelled out by the subtraction procedure. There was also no significant variation in the amplitude of the 300 Hz envelope component across all five stimuli; if sum waveforms included a cochlear microphonic component, it was consistent across frequency shifts and therefore could only have contributed to the results as a constant scaling factor that would not explain the frequency-shift-related changes found here.

**Pitch percepts and the behavior of distortion products**

Another potential complication results from the behavior of distortion products. Perceptual studies based on ‘hearing out’ distortion products, or on their interaction with interfering tones, indicate that perception of the 2f_1_ –f_2_ distortion product is not dependent on stimulus amplitudes, while the f_2_- f_1_ “envelope” distortion product is only perceived separately with stimuli above a certain amplitude level [23]. FFR experiments using two-tone stimuli (which induce weak unitary pitch percepts that rely on contextual manipulations and training [S3]), have found that some participants still show a response peak at the location of the “envelope” f_2_ – f_1_ neural distortion product (not generally perceived under these conditions) when the two tones are played to different ears, but none show signs of the 2f_1_ –f_2_ neural product, robustly obtained when the two tones are played to the same ear [17]. Experiments playing f_1_ and f_3_ to one ear and f_2_ to the other elicit the same missing fundamental pitch percepts as monaural presentations [1].

We propose that holistic pitch percepts emerge from the summation of envelope and fine-structure activity at higher frequencies (evoked neurally whether distortion products are separately perceived or not), while “hearing out” individual distortion products may require spatially separated peaks of activity, normally produced by the propagated component exciting the tonotopically appropriate region of the basilar membrane. This does not suggest that neural manifestations of distortion products are sufficient to explain all aspects of pitch perception. For example, auditory templates – experience-based mappings that mediate pitch perception based on the closest fit of stimuli to patterns instantiated in the brain – are a standard component of modern pitch theories [1, 3]. Since normally-developing organisms only rarely experience stimuli whose components are divided between the two ears, the neural signals measured here could play an important role in establishing experience-based pitch processing systems in the brain, which could then be called upon when trying to meaningfully parse stimulus patterns with unusual attributes. Further work examining FFRs with both monaurally- and dichotically-presented pitch complexes is required to more fully evaluate these ideas.

**References**

S1. Snyder RL, Schreiner CE (1984) The auditory neurophonic: basic properties. Hear. Res. 15: 261-280.

S2. Chimento TC, Schreiner CE (1990) Selectively eliminating cochlear microphonic contamination from the frequency-following response. Electroencephalogr. Clin. Neurophysiol. 75: 88-96.

S3. Smoorenburg GF (1970) Pitch perception of two-frequency stimuli. J. Acoust. Soc. Am. 48: 924-942.

**Figure Caption**

Figure S1. Mean evoked potential responses to stimuli. (a) The five different tone complexes used are shown color-coded above their constituent components on an amplitude – frequency plot (bottom), where the components of each tone are overlaid on a common set of axes. (b,c) Mean relative amplitude spectra (22 participants) for the 0° (in b) and 180° (in c) responses, showing the similarity in the response patterns evoked by these stimulus classes. Responses to each stimulus tone have been overlaid on the same set of axes. Colors refer to part (a) of the figure. (d,e) Mean relative amplitude spectra (n = 22 participants) of peaks in the fine-structure-related (difference waveform, d) and envelope-related (sum waveform, e) evoked potential responses. Responses to each stimulus tone have been overlaid on the same set of axes. Colors refer to part (a) of the figure.
