# Supplementary figures and images for: An Auditory Neural Correlate Suggests a Mechanism Underlying Holistic Pitch Perception
Source: PLoS One. 2007 Apr 11;2(4):e369. doi: 10.1371/journal.pone.0000369 (PMC1838520; doi:10.1371/journal.pone.0000369)

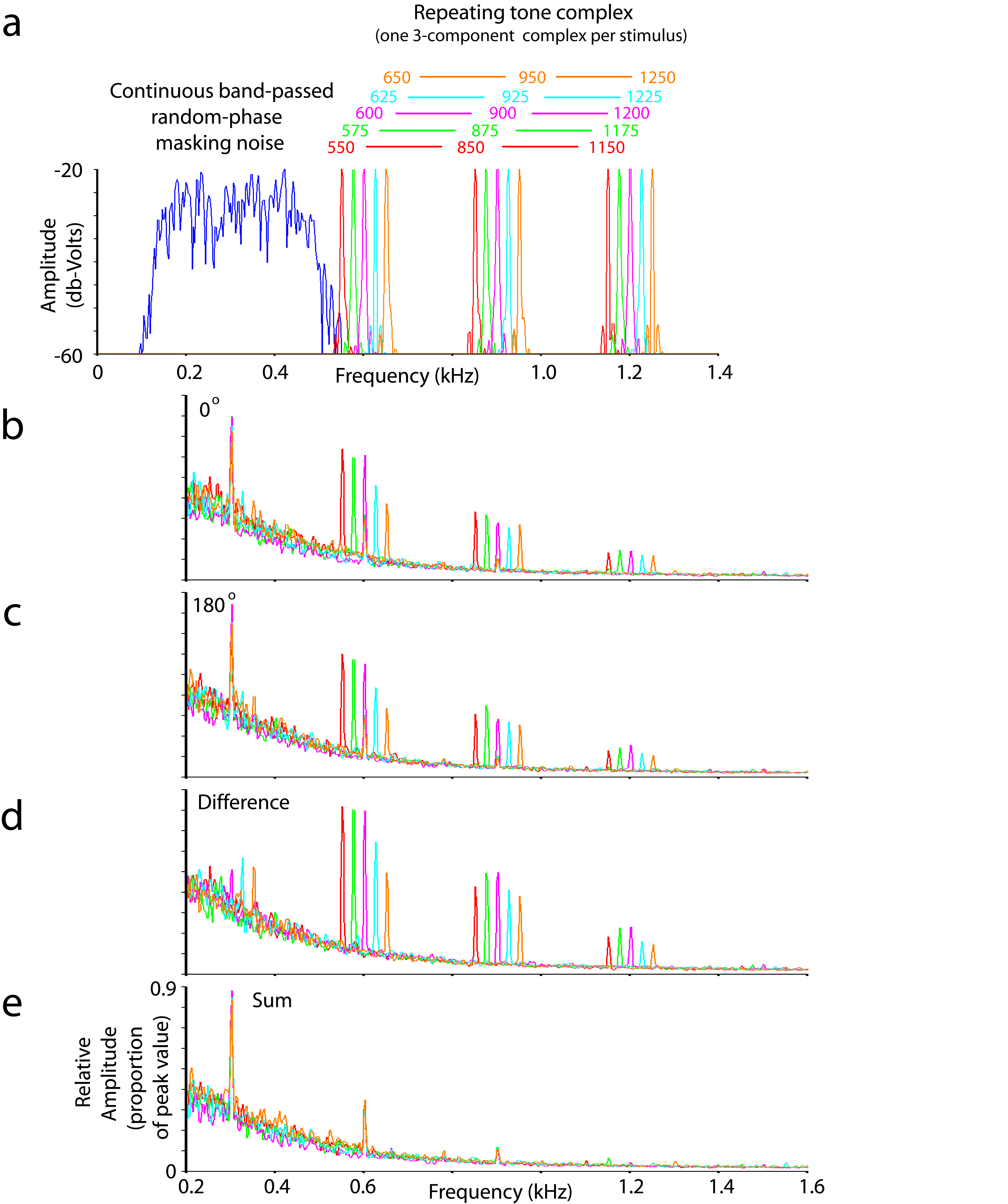

Supplement: Figure S1 — Mean evoked potential responses to stimuli. (a) The five different tone complexes used are shown color-coded above their constituent components on an amplitude - frequency plot (bottom), where the components of each tone are overlaid on a common set of axes. (b,c) Mean relative amplitude spectra (22 participants) for the 0° (in b) and 180° (in c) responses, showing the similarity in the response patterns evoked by these stimulus classes. Responses to each stimulus tone have been overlaid on the same set of axes. Colors refer to part (a) of the figure. (d,e) Mean relative amplitude spectra (n = 22 participants) of peaks in the fine-structure-related (difference waveform, d) and envelope-related (sum waveform, e) evoked potential responses. Responses to each stimulus tone have been overlaid on the same set of axes. Colors refer to part (a) of the figure. (2.27 MB TIF) [file pone.0000369.s002.tif]
